# Autonomic Dysfunction after Mild Traumatic Brain Injury

**DOI:** 10.3390/brainsci7080100

**Published:** 2017-08-11

**Authors:** Dmitry Esterov, Brian D. Greenwald

**Affiliations:** 1 JFK Johnson Rehabilitation Institute, Edison, NJ 08820, USA; 2 JFK Center for Head Injuries, JFK Johnson Rehabilitation Institute, Edison, NJ 08820, USA

**Keywords:** mild traumatic brain injury, mild TBI, concussion, autonomic dysfunction, dysautonomia, heart rate variability, graded exercise testing, post concussive syndrome

## Abstract

A mild traumatic brain injury (mTBI) is a complex pathophysiologic process that has a systemic effect on the body aside from solely an impairment in cognitive function. Dysfunction of the autonomic nervous system (ANS) has been found to induce abnormalities in organ systems throughout the body, and may contribute to cardiovascular dysregulation and increased mortality. Autonomic dysfunction, also known as dysautonomia, has been studied in moderate and severe TBI, and has emerged as a major contributing factor in the symptomatology in mTBI as well. Analysis of the ANS has been studied through changes in heart rate variability (HRV), pupillary dynamics, eye pressure, and arterial pulse wave in those with mild TBI. Graded exercise testing has been studied as both a method of diagnosis and as a means of recovery in those with mild TBI, especially in those with persistent symptoms. Given the studies showing persistence of autonomic dysfunction after symptomatic resolution of concussions, further research is needed to establish return to play protocols

## 1. Introduction

A concussion, or mild traumatic brain injury (i.e., mTBI) is a complex pathophysiologic process that is caused by traumatic biomechanical forces to the head [1]. There are an estimated 1.6–3.8 million sport-related concussions every year in the United States [2]. However, this likely underestimates the true incidence, as there are many mild traumatic brain injuries that go unreported or unrecognized [3]. The term concussion is synonymous with mTBI, however, clinicians may use the term concussion to describe instances in which an individual experiences transient alteration in mental status from a sports related head injury, whereas mTBI is used in the general medical context [4]. Overall, terms such as concussion, mild head injury, and mTBI are often used interchangeably to describe the physical injury itself, as well as its immediate and later symptomatic consequences [5].

Mild TBI results in a constellation of physical, cognitive, vision, emotional, and sleep-related disturbances. Signs and symptoms are broad and include headache, dizziness, gait disturbance, nausea, vomiting, photophobia, trouble focusing, and fatigue. A person with mTBI may have slowed mental processing, concentration deficits, memory impairment, irritability, and depression [6,7]. Symptoms may last from several minutes to days, weeks, months or even longer in some cases. When symptoms of concussion persist beyond three months, these symptoms become characterized as post-concussive syndrome. Symptoms usually fully resolve, however, approximately 15% of people can have long-term sequalae [6,7].

Traumatic brain injury severity can be diagnosed and classified as mild, moderate, or severe based on certain measures including the duration of loss of consciousness, post traumatic amnesia (PTA), and initial Glasgow Coma Scale (GCS) score (Table 1). A mild traumatic brain injury is defined as an injury with Glasgow Coma Scale of 13–15 with loss of consciousness less than 30 min and pos- traumatic amnesia less than 24 h (Table 1). A moderate TBI is defined as having a GCS score of 9–12, and a severe TBI as a GCS score of 8 or below [6]. The term mild traumatic brain injury may be misleading as it includes a range of manifestations from both mild symptoms to persistent and ongoing disability [6,8].

In the majority of people, mTBIs are not associated with macroscopic abnormalities on imaging. Diffuse axonal injury is a primary pathological feature of all types of traumatic brain injury, which may be seen on CT or MRI by petechial hemorrhages [6]. The injury can occur with both contact forces to the head, as well as rapid acceleration/deceleration head movements [6]. Pathophysiology of concussion on a molecular level includes changes to cell membrane permeability, ion transport regulation, neurotransmitter release, cellular metabolism, and changes to cerebral blood flow [2]. Acutely, there is a release of excitatory neurotransmitters resulting in widespread neural depolarization and ion imbalances, which leads to an upregulation of adenosine triphosphate-dependent sodium-potassium pumps and increased cellular glucose demands. After the initial period of hypermetabolism, mitochondrial dysfunction leads to decreased glucose utilization and metabolism [10]. In patients with TBI, there is impairment in cerebral blood flow, where cerebral hypoperfusion leads to further injury [2].

## 2. Autonomic Nervous System in TBI

Dysfunction in the autonomic system has been found to be a major factor in the symptomatology in TBI, including in mTBI. The central autonomic network is a complex network in the central nervous system (CNS) involving the cerebral cortex (the insular and medial prefrontal regions), amygdala, stria terminalis, hypothalamus, and brainstem centers (periaqueductal gray, parabrachial pons, nucleus of the tractus solitarius, and intermediate reticular zone of the medulla) [11]. The relationship between the prefrontal cortex of the brain and the heart has been studied and the amygadala is believed to be the major efferent source of modulation of autonomic, endocrine, and cardiovascular responses [8,12,13]. New areas of research also indicate that the frontal cortex can modulate vagal and myogenic tone [14]. The autonomic nervous system has two divisions: the sympathetic nervous system (SNS) and the parasympathetic nervous system (PNS). The sympathetic nervous system (SNS) is predominantly involved in cardiac and vascular regulation and in “fight or flight” conditions and the parasympathetic nervous system (PNS) has a smaller influence on the peripheral vasculature and is active in more quiet conditions [11]. Postganglionic sympathetic fibers innervate the atria, the ventricles, and coronary arteries from the cervical ganglia as the superior, middle, and inferior cardiac nerves or from thoracic ganglia at the TI–T4 level. Stimulation causes increased heart rate, increased myocardial contractility, and coronary vasodilatation.

The ANS functions without conscious voluntary control. The “fight or flight” response of the sympathetic system is a whole body response that includes a release of epinephrine and norepinephrine from the adrenal medulla, as well as a widespread vasoconstriction in the body. The PNS, on the other hand, decreases the heart rate, which helps to conserve energy under resting conditions. The ANS is not only involved in vascular and cardiac regulation, but because it innervates cardiac muscle, smooth muscle, and various endocrine and exocrine glands, it influences the activity of most tissues and organ systems in the body. The regulation of blood pressure, gastrointestinal responses, contraction of the urinary bladder, focusing of the eyes, and thermoregulation are all controlled by the ANS [11].

Dysfunction of the autonomic nervous system in general has been found to induce abnormalities in organ systems throughout the body. Systemic complications after TBI may occur because of neurogenic causes such a large catecholamine release and inflammatory response after the injury [15]. Cardiac sequelae of an injury may produce arrhythmias, ischemia, or a myocardial infarction. Overall, a brain injury may cause various systemic abnormalities from increased sympathetic activity leading to immune system depression [16]. Endocrine abnormalities may happen, as well as with pathology to the hypothalamic-pituitary axis. Autonomic dysfunction, which is also known as dysautonomia, has been found to correlate in patients with irritable bowel syndrome [17]. There have also been correlations found between the ANS and depression, as well as in psychiatric disorders. Substantial reductions in heart rate variability (HRV) have been seen across psychiatric disorders, and these effects remained significant even in medication-free individuals [18]. HRV changes have been found to be a marker for generalized anxiety disorder, post-traumatic stress disorder, or a general predisposition to react sympathetically to external or internal stressors [18]. Dysregulation of the autonomic nervous system is a common characteristic in depression, schizophrenia, and panic disorders, as well [18].

In general, autonomic dysfunction in TBI is a significant pathophysiological mechanism that can lead to a worsened quality of life. The presence of autonomic dysfunction has also been shown to correlate with increased morbidity and mortality in moderate and severe TBI [19]. A decrease in baroreflex sensitivity, a measure of ANS activity, has been shown to correlate with an increased risk of cardiac complications, including an increased risk of arterial hypertension [19]. Perturbations of the ANS and its imbalance consisting of either increased sympathetic or reduced vagal activity may result in ventricular tachyarrhythmias and sudden cardiac death [19]. In general, moderate/severe TBI patients who have autonomic dysfunction have a longer duration of posttraumatic amnesia, longer admissions to the hospital, and greater overall health costs [20].

## 3. Assessment

In the study of the relationship between mTBI and the autonomic system, various modalities have been used. Among the available noninvasive techniques for assessing the autonomic status, measuring heart rate variability (HRV) has been commonly used. Heart rate variability is a noninvasive electrocardiographic marker reflecting the activity of the sympathetic and vagal components of the ANS on the sinus node of the heart. It expresses the total amount of variations of both instantaneous heart rate as well as RR intervals (intervals between QRS complexes of normal sinus depolarization). Thus, HRV analyses the tonic baseline autonomic function. Recording of HRV may generally be performed on the basis of 24 h Holter monitor or on shorter periods ranging from 0.5 to 5 min. There are two standard metrics for measuring HRV—a time domain (changes over time) and a frequency domain (measurement of a spectrum of oscillatory components of the heart). The standard deviation of the normal to normal interval (SDNN) is one of the most commonly-used statistical methods, which looks at the standard deviation of the R-to-R interval (HRV interventions for concussion and rehabilitation—Condor). Measurements consist of low-frequency (LF) and high-frequency (HF) bands, where the ratio between the LF and HF components is accepted as an important marker of sympathovagal balance [21].

In a healthy person, there will be continuous physiological variations of the sinus cycles reflecting a balanced sympathetic and vagal state and normal HRV. In a damaged heart which suffered from myocardial necrosis, the changes in activity in the afferent and efferent fibers of the ANS and in the local neural regulation will contribute to the resulting sympathovagal imbalance reflected by a diminished HRV [21]. In general, greater HRV suggests that the ANS is appropriately responding to the requirements of the environment, whereas lower HRV suggests that the ANS is not modulating the heart rate as efficiently [21]. Based on established clinical data, a decreased global HRV is considered a strong predictor of increased all-cause cardiac and/or arrhythmic mortality, particularly in patients at risk after MI or with CHF [8].

Changes to HRV have been found in those with mTBI. Some studies have found abnormalities in HRV in those with concussions both at rest, with isometric handgrip exercise, and with aerobic exercise, while other studies have failed to find these changes at rest or during isometric handgrip exercise [22]. A study examined these parameters in the post-acute stage in athletes approximately three months post-injury and found changes in autonomic function as compared to control athletes [21]. HRV disturbances post-concussion in athletes have been found to persist past symptom resolution and return to play protocols as well [23]. A systematic review of measuring the effect of concussion on cardiac autonomic function through HRV did show evidence to suggest that cardiac autonomic function is altered during physical activity after a concussion [24]. There was conflicting evidence regarding cardiac autonomic function at rest following concussion. This review analyzed nine full length articles and four abstracts, and concluded that the current literature is somewhat limited by small sample sizes, lack of pediatric or female participants and lack of follow up [24].

There have been other measures that have studied the link between concussion and autonomic dysfunction, including measurements of the pupillary light reflex, the arterial pulse wave (APW), eyeball pressure, as well as graded exercise testing. Studies have shown that the pupillary light reflex is slowed in mTBI, in that they have found a decrease in average pupillary constriction velocity and average dilation velocity as compared with normal individuals [25]. The arterial pulse wave (APW) has a distinct morphology whose contours reflect dynamics in cardiac function and peripheral vascular tone as a result of sympathetic nervous system control [26]. A study analyzed changes in APW at rest and during initial isometric handgrip in concussed athletes and non-injured controls. Use of APW showed that the study group had an increase in peripheral artery stiffness. This was thought to be an adaptation to a decline in stroke volume during transition from a rest state to a state of increased metabolic demand within 48 h of concussion [26]. Cardiovascular responses to forced breathing, standing, and the Valsalva maneuver after concussion were studied as well, indicating large acute increases in resting SBP, HR, and SBP perturbations during standing [21]. Another study analyzed purely parasympathetic activation that occurs independently from baroreceptive pathways through eyeball pressure (EP) stimulation [27]. This study used EP to evaluate cardiovascular responses after mTBI, demonstrating abnormal cardiovascular responses to the parasympathetic stimulus in the study group [27]. Graded exercise testing has also emerged as a way of studying the connection between the autonomic system and concussion through measuring blood pressure responses and heart rate. This has been measured through the work of John Leddy and Karl Kozlowski, who showed patients with symptoms of post-concussion syndrome had a symptom-limited response to exercise [28].

## 4. Treatment

Current treatment of those with mTBI has been physical and cognitive rest, as about 80–85% of patients with a concussion will recover to their neurological baseline within 1–2 weeks with this treatment [2]. One study measured the recovery of NCAA football players after a concussion, finding that ninety-one percent of players with concussion returned to personal baseline symptoms within seven days of concussion [29]. In the athletic population, patients with concussions are withheld from sporting activities until they are asymptomatic. Once asymptomatic at rest, they are treated with a return-to-play protocol progressing from light aerobic activity, sport-specific exercise, non-contact training drills, full-contact practice and, finally, a return to play (Table 2). In this case, patients progress through each stage as long as they are asymptomatic in the previous stage for 24 h until they are back to full contact sporting events. For patients with significant symptoms as rest, this resting approach will result in symptom improvement in the majority of patients [30].

Patients who do not recover after three months are categorized as having post-concussive syndrome (PCS), which affects about 15% of patients [6,7]. Symptoms include persistent headaches, depression, difficulty concentrating, dizziness, imbalance, and fatigue. Risk factors for PCS include premorbid psychiatric illness, prior migraine headaches, female gender, and a younger age [31]. Like concussion, PCS is a clinical diagnosis without a diagnostic biomarker or neuroimaging findings. In this case, guidelines for management have not been established, and patients are generally recommended to continue physical and cognitive rest [2].

Leddy and his colleagues have studied treatment of PCS through looking at distinct subtypes of PCS based on unique features of the physical examination, clinical history, and graded exercise testing [2]. These subtypes are referred to as post concussive disorders (PCD) and can be broken down as physiological post-PCD, vestibulo-ocular PCD, and cervicogenic PCD. Physiological PCD is characterized by concussion symptoms from alterations in cerebral blood flow secondary to autonomic nervous system dysfunction. Vestibulo-ocular PCD is characterized by symptoms secondary to dysfunction of the vestibular and oculomotor systems, and cervicogenic PCD is characterized by muscle trauma and inflammation secondary to cervical spine somatosensory system. It is also important to differentiate patients who have clinical depression and post traumatic mood disorders, and those that have symptoms secondary to migraine headaches.

Graded exercise testing has been studied as a method to safely and reliably diagnose physiologic/autonomic dysfunction in concussion from other subtypes. The graded exercise test, also known as the Buffalo Concussion Treadmill Test, is based upon the Balke cardiac treadmill test, which has shown to be safe in cardiac and orthopedic patients [32]. Absolute contraindications include an increased risk for cardiopulmonary disease, which include individuals with known cardiovascular, pulmonary, or metabolic disease, signs, and symptoms suggestive of cardiovascular or pulmonary disease, or individuals aged greater than forty-five who have cardiac risk factors. These include a family history of myocardial infarction, coronary revascularization, or sudden death before age 55. The graded treadmill test looks at the rate of perceived exertion, as well as the Borg scale. Heart rate and blood pressure are measured and the incline level increases. The test is stopped at a significant exacerbation of symptoms [32].

**Figure 1:**
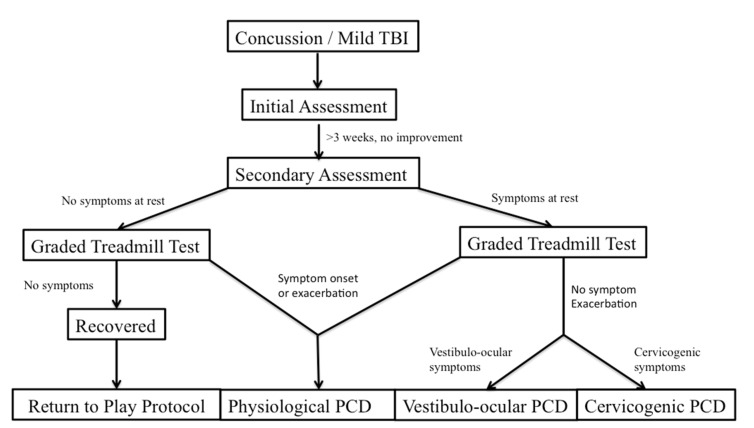
Proposed algorithm for diagnosing post-concussive disorder subtypes based on graded treadmill testing [2].

The patient’s performance and symptom pattern during the BCTT, combined with a pre-test physical examination, can help with the differential diagnosis of PCD (Figure 1). Patients with physiologic PCD, which is related to autonomic dysfunction, will typically have an exacerbation of symptoms during the graded exercise test, and will have to stop the test early. On the other hand, patients with cervicogenic PCD, vestibulo-ocular PCD, as well as those with posttraumatic mood disorders and migraines should not have symptom exacerbation when they perform the BCTT [32]. From there, further clinical history as well as physical examination can help differentiate some of the pathophysiologic mechanisms and guide treatment more precisely.

While the main recommendation for treatment of PCS has consisted primarily of physical and exercise restriction, the physical deconditioning that accompanies rest is also significant, as well. Bed rest can reduce cerebral blood flow for a substantial period of time, and prolonged physical rest may lead to deconditioning and cardiovascular declines [33]. Thus, a lack of physical exercise could hinder, rather than aid, recovery in those with prolonged symptoms in those with autonomic dysfunction. There are studies that have looked at submaximal exercise as a treatment option for those with persistent symptoms related to autonomic dysfunction after a concussion. Leddy et al. compared the outcomes of 12 PCD patients who were symptomatic at rest and who experienced symptom exacerbation during graded treadmill testing. All patients were prescribed aerobic exercise for the same duration they achieved during treadmill testing but at 80% of the maximum heart rate achieved, once daily, 5–6 days a week, with use of a digital heart rate monitor. Patients were instructed to have another person supervise them during exercise. After treatment for three weeks, all patients reported improved symptoms as well as improved peak heart rate without symptom exacerbation [2]. Lal et al. performed a systematic review on the effect of physical exercise after concussion. Fourteen studies met the inclusion criteria, and physical exercise was found to have a statistically significant improvement in the post-concussion symptom scale (PCSS) score and symptoms in patients with a concussion [34].

## 5. Conclusions

There is evidence of ANS dysfunction after a mild TBI, studied through various mechanisms, including changes in heart rate variability, arterial pulse wave analysis, graded exercise testing, and pupillary dynamics. Though studies have shown changes to autonomic function in the post-acute stage after a concussion [19], further prospective studies are needed to understand the long term effects of mild TBI on the autonomic nervous system, and the possible systemic effects of ANS dysfunction in mild TBI. Larger prospective studies are needed to determine whether graded exercise testing and submaximal exercise in patients with persistent symptoms will be a standard of care in management of concussions. Given the studies showing persistence of autonomic dysfunction after symptomatic resolution of concussions, further research is needed to evaluate and more accurately assess return-to-play protocols. Mild TBI is a significant health problem that affects athletes, civilians, and military populations, and can significantly impact the life of those affected.

## Figures and Tables

**Table 1:** Diagnostic criteria for a mild traumatic brain injury (Mild Traumatic Brain Injury Committee, Head Injury Interdisciplinary Special Interest Group, American Congress of Rehabilitation Medicine) [9].

Diagnostic Criteria for a mild TBI:
A traumatically induced physiological disruption of brain function, as manifested by at least one of the following
Any loss of consciousness
Any loss of memory for events immediately before or after the accident
Any alteration in mental state at the time of the accident (e.g., disoriented, confused)
Focal neurological deficit that may or may not be transient but where the severity of the injury does not exceed the following: Loss of consciousness approximately 30 min or less After 30 min, an initial Glascow Coma Scale of 13–15 Post Traumatic Amnesia not greater than 24 h

**Table 2:** Graduated return to sport strategy after a concussive injury [1].

Rehabilitation Stage	Activities
(1) No Activity	Complete Physical and Cognitive Rest
(2) Light aerobic Activity	Walking, exercise Bike
(3) Moderate Activity	Moderate jogging, weight lifting
(4) Heavy, non-contact activity	Non-contact sport-specific drills
(5) Practice and full contact	Full Contact Practice
(6) Competition	Game Play
